# An Energy-Efficient Game-Theory-Based Spectrum Decision Scheme for Cognitive Radio Sensor Networks

**DOI:** 10.3390/s16071009

**Published:** 2016-06-30

**Authors:** Shelly Salim, Sangman Moh

**Affiliations:** 1 School of Engineering Mastership, Pohang University of Science and Technology, 77 Cheongam-ro, Nam-gu, Pohang, Gyeongbuk 37673, Korea; shsalim@postech.ac.kr; 2 Deptartment of Computer Engineering, Chosun University, 309 Pilmun-daero, Dong-gu, Gwangju 61452, Korea

**Keywords:** cognitive radio, cognitive radio sensor network, energy-efficiency, game theory, spectrum decision

## Abstract

A cognitive radio sensor network (CRSN) is a wireless sensor network in which sensor nodes are equipped with cognitive radio. In this paper, we propose an energy-efficient game-theory-based spectrum decision (EGSD) scheme for CRSNs to prolong the network lifetime. Note that energy efficiency is the most important design consideration in CRSNs because it determines the network lifetime. The central part of the EGSD scheme consists of two spectrum selection algorithms: random selection and game-theory-based selection. The EGSD scheme also includes a clustering algorithm, spectrum characterization with a Markov chain, and cluster member coordination. Our performance study shows that EGSD outperforms the existing popular framework in terms of network lifetime and coordination overhead.

## 1. Introduction

A wireless sensor network (WSN) is a network of a large number of densely deployed sensor nodes [1,2] that are able to monitor various ambient conditions. Typically, a WSN operates using the unlicensed spectrum band. As time has gone by, the unlicensed band usage has become very crowded. Meanwhile, several field measurements showed low utilization on licensed spectrum bands [3,4,5]. The unbalanced situation makes the overall spectrum utilization low.

To improve spectrum utilization and to accommodate users in the unlicensed spectrum band, an idea arose: allow unlicensed devices/users to transmit in the licensed band. In this environment, there are two types of users in the licensed bands: primary users (PUs) and secondary users (SUs). SUs need transmission devices capable of detecting PU transmissions, operating on a wide range of spectrum bands, and switching their operating spectrum/channel. Cognitive radio (CR) supports those capabilities and more.

Since its introduction in 1999 [6], cognitive radio (CR) technology has received a lot of attention. The term “cognitive radio” first emerged in [7]; however, the definition of CR adopted here follows the thorough description by Haykin in [8] in agreement with the more general description by the FCC [9]. Many CR features and key implementation issues in existing wireless networks have been studied extensively [10,11,12,13]. International standardization bodies are developing standards to guide the implementation of CR [14].

In this study, we are interested in the integration of CR in a wireless sensor network, i.e., a cognitive radio sensor network (CRSN) [15]. We believe that a wireless sensor network will be one of the fundamental elements in realizing the Internet of Things (IoT) [16] and a “smarter world” [17]. In CRSNs, the SUs are the sensor nodes.

The main goal of implementing CR in WSNs is to improve the spectrum utilization of unlicensed spectrum bands [18,19]. If the PUs share the licensed spectrum band with SUs, then the spectrum utilization would be increased [20]. This situation is called inter-spectrum sharing, which can be supported by setting a rule that the SUs must not interfere with the PU activity. However, SUs also have to share the vacant channels between them, that is intra-spectrum sharing. Intra-spectrum sharing can be achieved by applying an effective spectrum decision.

A spectrum decision selects the operating channel of the SU among a number of possible channels [21]. The fundamental requirement of the spectrum decision is to select an operating channel that is reported vacant by the spectrum sensing [22,23,24]. When a single SU is considered, a spectrum decision may choose the “best” channel (highest signal-to-noise ratio, lowest bit error rate, and so forth) as its operating channel. However, in a CRSN network with multiple SUs, to select the best channel as the operating channel does not turn out to be the best solution. In this paper, we propose a spectrum decision scheme for CRSNs, called an energy-efficient game-theory-based spectrum decision (EGSD) scheme. In the proposed work, game theory is applied to the spectrum selection algorithm because it considers the interactions between multiple decision-makers (in this case, the SUs).

However, a spectrum selection algorithm alone is not sufficient for a CRSN. The proposed spectrum decision scheme contains not only two spectrum selection algorithms but also a number of supporting algorithms. In this paper, we propose an energy-efficient game-theory-based spectrum decision (EGSD) scheme for CRSNs to prolong the network lifetime. More specifically, we present: (1) a spectrum decision scheme for a CRSN; (2) two types of spectrum decision algorithm, i.e., random selection and game-theory-based selection; and (3) simple yet effective supporting algorithms for clustering, spectrum sharing, and spectrum access. According to the simulation results, the proposed EGSD has 46.6% longer lifetime and 31.7% lower overhead compared to the conventional minimum-variance-based spectrum decision (MVSD) scheme.

The rest of this paper is organized as follows: we provide some related works in Section 2. In Section 3, we describe the details of the proposed EGSD scheme and the two spectrum selection algorithms: random selection (EGSD-R) and game-theory-based selection (EGSD-G). The performance evaluation of the proposed scheme is described in Section 4, for both spectrum selection algorithms and a representative related work. Finally, we conclude our paper in Section 5.

## 2. Related Works

To the best of our knowledge, at the time we proposed this scheme, there was no exact related work in the field of spectrum decision method for CRSNs. Nevertheless, there are spectrum decision methods proposed for general/other types of CR networks.

Surveys on spectrum decision in cognitive radio networks and CRSNs are presented in [25] and [26], respectively. Several works deal with spectrum-related management for CRSNs. New parameters to support channel assignment are proposed in [27,28], where they use an R-coefficient to represent predicted residual energy, and the value of the spectrum usability to represent the spectrum idle rate and spectrum quality, respectively. Both works showed improved performance, but one work lacks what the other work covered: a method that is energy-aware as well as spectrum-aware would be preferred. The channel assignment is combined with routing in [29] with packet-based channel assignment. While the consideration of routing may be one of the appeals of this work, it is not an essential issue for a CRSN. Thus, this work may be suitable for very large-scale CRSNs. A similar drawback can be inferred from grid-based channel assignment [30]. The Markov decision process is adapted in channel allocation [31] and operation mode selection [32]. The Markov decision process and game theory are both decision-making algorithms in which the decision makers interact with opponents in a dynamic environment. However, as discussed in [33,34], game theory is considered more suitable than the Markov decision process in an environment with multiple decision makers, where their actions contribute to the dynamics of the environment. A centralized spectrum allocation based on game theory is proposed in [35]. While a centralized approach is plausible because there is at least one sink in a CRSN, it does not cope well with spectrum heterogeneity over time and space. The result of centralized spectrum allocation may not be optimum for every sensor node, not to mention the additional overhead cost involved.

There are some works on spectrum decision methods for usual cognitive radio networks (except for CRSNs). An analysis of load-based, interference-based, and joint load- and interference-based decision schemes showed that basic metrics were able to improve the performance of cognitive ad hoc networks, though only in certain cases [36]. Novel parameters for spectrum decision were proposed, such as a request index to represent the quality of service requirements of SUs [37], channel usage state [38], and outage probability [39]. While those works that used new parameters showed improved performance, the definition of parameters was rather fixed (channel usage state value was predicted based on its history, but the storage was predetermined and limited.) Online learning was used in a spectrum decision algorithm based on predictions; however, the focus was merely to calculate the probability of handover [40]. Similarly, specific purpose spectrum decision methods are proposed in [41,42,43], in which they were focused on time minimization and security issues (the last two works). An energy-aware spectrum decision framework is proposed in [44], where it includes an energy-monitoring unit with a predefined threshold.

A spectrum decision framework for a general CR network, which is proposed in [45], is chosen to be the comparison work for our proposed framework, EGSD. The reasons are that: (1) it is similar to EGSD in that the comparison work is a framework (instead of a standalone algorithm or protocol) and (2) it has been highly cited by other papers. Moreover, the comparison work is a complete and detailed work with strong numerical analysis and performance evaluation results. The comparison work introduced two algorithms under its spectrum decision block: minimum-variance-based spectrum decision (MVSD) and maximum-capacity-based spectrum decision (MCSD). We only compared EGSD with MVSD because MVSD was designed to support real-time applications, which is the nature of CRSN applications. We also exclude the admission control function because, in our network scenario, there will be no new sensor nodes installed in the middle of the network operation. MVSD is applied in an infrastructure-based CR network in which the base station performs the spectrum decision (centralized approach). The CR nodes or sensor nodes perform spectrum sensing, send the results to the base station, and wait for the spectrum decision results. Interesting readers may refer to reference [45] for more details.

## 3. Energy-Efficient Game-Theory-Based Spectrum Decision

We propose an energy-efficient distributed spectrum decision (EGSD) scheme for a CRSN. Firstly, we present the CRSN settings, models, operation modes, and notations that we used. Then, we describe the modules of the EGSD scheme on the coordination mode and data transmission mode.

### 3.1. CRSN Settings

The EGSD scheme is proposed for CRSNs where there is a number of sensor nodes placed randomly in an area of interest and a sink located at the center of the area. The CRSN is able to access three spectrum bands: television (TV), ISM 2.4 GHz, and ISM 5 GHz. The TV band consists of 30 channels [46], with the first channel being the common control channel. The CRSN operations, management and transmission activities, are performed in frames. A frame is equal to 2 s, and it is divided into 111 time slots where each time slot equals 18 ms [47] and the last time slot is equal to 20 ms.

The CRSN is located in a populated city where the incumbent users of the TV band (herein PU) exist. The PU model is as follows: The maximum number of PUs is predetermined, however the number of PUs at a certain time is not fixed. The PUs can be either active or passive. A PU is active when it transmits or receives a data transmission; otherwise, it is passive. A passive PU can become active, and vice versa. PUs can change their operating channel and move their location while they are active.

The licensed and unlicensed channel models are as follows: the sensor nodes perform spectrum sensing in licensed channels, and they obtain information on whether the channel is vacant. The unlicensed channel may be occupied by other wireless networks. Thus, we model the unlicensed channel in terms of peak interferences. The peak interferences take place on a certain channel, and they affect up to two adjacent channels. When a sensor node selects an unlicensed channel with high interference, the probability of transmission failure is higher, whereas when it uses a licensed channel, the transmission success is guaranteed unless the total number of sensor nodes transmitting on that channel is higher than a certain threshold. If the number of total sensor nodes transmit-ting on a licensed channel exceeds the threshold, then the probability of transmission failure increases with the number of channel occupants. We define one licensed channel as the common control channel (CCC). The CCC is used exclusively to transmit control packets, and the channel’s occupant threshold is higher than the threshold for other licensed channels.

Each sensor node is equipped with one CR transceiver; thus, it is able to tune in one channel at a time. Meanwhile, the sink has two CR transceivers, in which one transceiver is always tuning in the CCC and another can switch its channel. The sink has a constant power supply and higher computation capability. It can broadcast its control packets on the CCC so that every sensor node can receive them. Fundamentally, enabled by the CR’s reconfiguration ability, the sensor nodes are also able to transmit to the sink directly. However, direct transmission is not favorable because long-distance transmission requires high energy consumption. We designed the EGSD scheme for CRSNs to suit environmental maintenance systems that require periodic data collection.

**Figure 1:**
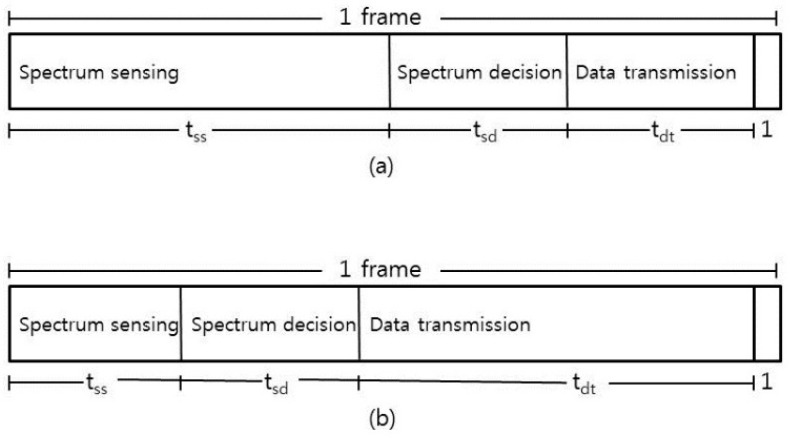
The modules of (**a**) C mode and (**b**) D mode.

The EGSD scheme has two operation modes: coordination mode (C mode) and data transmission mode (D mode). A frame can be in either C mode or D mode. In C mode, coordination activities take most parts of the frame, whereas in D mode, environment-sensing data collections are encouraged. Both modes consist of the same modules: spectrum sensing, spectrum decision, and data transmission module. The duration of spectrum sensing module (*t_ss_*) is maximized in C mode to support full spectrum sensing, and it is minimized in D mode where only partial spectrum sensing is performed. On the other hand, the duration of data transmission module (*t_dt_*) is maximized in D mode. The duration of the spectrum decision module (*t_sd_*) for both modes is the same (see Figure 1).

The operation mode of the initial frame is C mode, and the operation mode of the following frames is decided by the sink. The last time slot in each frame is allocated to sensor nodes to report to the sink whether they require C mode or not through the CCC. There are two types of C-mode request: normal request and urgent request. When the sink receives at least one urgent request, it decides the operation mode of the next frame will be a C mode, and broadcasts this information on the CCC. When the sink receives normal requests, it does not immediately set the next mode to be a C mode, but waits until the number of frames associated with normal requests has exceeded a certain threshold. If there is no request at all, then the next mode is set to be D mode. The conditions in which a sensor node requests C mode are explained in the next section. Whenever a C mode is performed, the previous clustering topology is resettled.

In the proposed EGSD scheme, we use the following notations:

- A set of *N* sensor nodes acting SUs *S* = {*S_1_*, *S_2_*, …, *S_N_*} with *status*(*S_i_*) ∈ {*active*, *passive*} and *class*(*S_i_*) ∈ {*CH*, *CM*, *SN*}, 1 ≤ *i* ≤ *N*. (Players)
- A set of *M* primary users *T* = {*T_1_*, *T_2_*, …, *T_M_*} with *status*(*T_j_*) ∈ {*active*, *passive*}, 1 ≤ *j* ≤ *M*. (Players)
- A set of (*L*+1) licensed channels *A* = {*A_0_*, *A_1_*, *A_2_*, …, *A_L_*} and its SNR observed by *S_i_* *SNR_i_* = {*SNR_1_*, *SNR_2_*, …, *SNR_L_*}.
- A set of *K* unlicensed channels *B* = {*B_1_*, *B_2_*, …, *B_K_*} and its SNR observed by *S_i_* *SNR’_i_* = {*SNR’_1_*, *SNR’_2_*, …, *SNR’_K_*}.
- A common control channel *C_control_* = *A_0_*.
- An operating channel of *S_i_* on current frame *f* = (*C_i_*)*_f_* = *A_x_* or *B_y_* or Ø, where 1 ≤ *x* ≤ *L*, 1 ≤ *y* ≤ *K*, and Ø means empty set.
- A backup channel of *S_i_* on current frame *f* = (*C’_i_*)*_f_* = *A_x_* or *B_y_* or Ø, where 1 ≤ *x* ≤ *L*, 1 ≤ *y* ≤ *K*, and (*C’_i_*)*_f_* ≠ (*C_i_*)*_f_*.
- *Status*(*A_x_*)*_i_* ∈ {*available*, *not* *available*, *obsolete*, *idle*, *busy*}, 1 ≤ *x* ≤ *L*, observed by *S_i_*.
- *Status*(*B_y_*)*_i_* ∈ {*clean*, *noisy*, *unknown*}, 1 ≤ *y* ≤ *K*, observed by *S_i_*.
- CH type = *type*(*CH*)*_i_* ∈ {0, 1, 2, 3, 4}, *class*(*S_i_*) = (*CH*), 1 ≤ *i* ≤ *N*.

### 3.2. Coordination Mode (C Mode)

#### 3.2.1. Spectrum Sensing Module of C Mode

The spectrum sensing module of C mode exploits full spectrum sensing (FSS), in which the sensor nodes perform spectrum sensing on entire licensed channels. The sensor nodes sleep for a random duration before they start to perform spectrum sensing. The main purpose of random delay is to support the clustering algorithm. The random delay is brief, and its maximum duration is predetermined.

To prevent false detection of PU transmissions, all sensor nodes must not perform any data transmission during FSS (quiet period). The sensor nodes obtain the information of whether there is an ongoing transmission on a channel, and record the channel’s SNR. If there is no PU transmission detected by sensor node *S_i_* on licensed channel *A_x_*, then:

(1) $$Status{\left(A_{x}\right)}_{i}=(available)$$

otherwise:

(2) $$Status{\left(A_{x}\right)}_{i}=(not\;available)$$

The outcomes of FSS are a list of all licensed channels, their status, and the variables to calculate SNR. Next, the sensor nodes continue to perform clustering.

#### 3.2.2. Clustering Algorithm of C Mode: Residual-Energy-Based Clustering

The EGSD scheme also includes a simple clustering algorithm, called residual-energy-based clustering because we favor the energy-efficient clustered topology over direct transmission. However, we avoid a sophisticated clustering algorithm despite its performance improvement because, in CRSNs, there are additional energy-consuming spectrum-related activities compared to traditional wireless sensor networks. We try to minimize energy consumption by proposing a simple clustering method.

**Figure 2:**
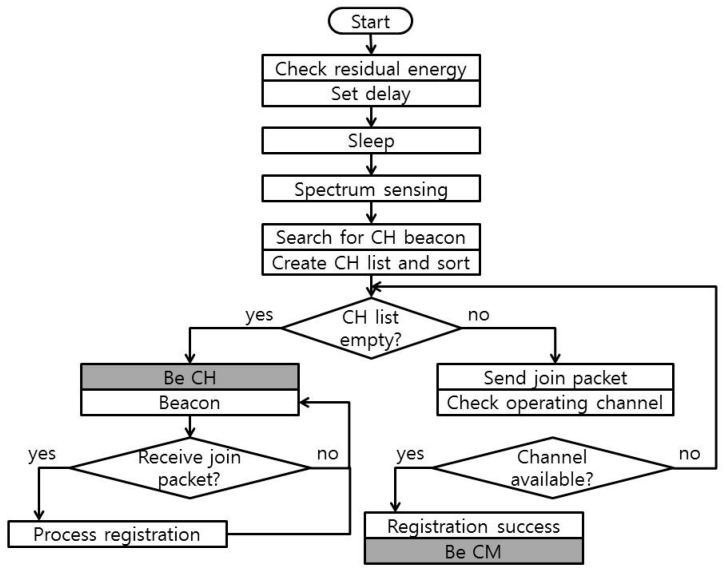
Residual-energy-based clustering.

The clustering algorithm is triggered on the C mode only. Some parts of the clustering algorithm start before spectrum sensing, and the rest begin after spectrum sensing, as illustrated in Figure 2.

Initially, all sensor nodes are in the class sensor nodes, class(*S_i_*) = (*SN*), *∀i*. The sensor nodes check the residual energy and set the maximum limit of the delay based on it. If the residual energy is high, then the maximum limit of the delay is low, and vice versa. The actual delay duration is set by following a uniform distribution with zero and the previously settled maximum limit. The sensor nodes go to sleep during this delay and wake up to perform FSS. The sensor node *S_i_* listens on the *C_control_* for CH beacons, creates a *CH_list* = {*CH_1_*, *CH_2_*, …, *CH_r_*}*_i_*, and sorts the *CH_list* based on the received signal strength indicator (RSSI) in a descending manner. Here, we would like to explain the reason for adapting a random delay: without a random delay, every sensor node would perform the same activity at the same time, which would result in no CH found when they try to find a CH because every sensor node is currently finding a CH. Later, when each of them decides to become a CH, probably no node is looking for a CH anymore because all of them have also become CHs.

The sensor nodes try to join the CH on the top of the list first. The cluster registration method is as follows: the sensor node sends a join packet to the cluster head, and the cluster head replies with operating channel information. The sensor node checks whether the assigned operating channel is idle on its side (spectrum sensing result might be different spatially.) If the assigned operating channel is available, then the sensor node *S_i_* becomes a CM of that CH, i.e., *class*(*S_i_*) = (*CM*), sets its operating channel as the assigned operating channel, and goes into sleep state until the end of the spectrum sensing module. If the assigned operating channel is not available, then the sensor node checks the CH list and tries the next CH on the list, given that the remaining time is sufficient. Otherwise, the sensor node becomes a CH, i.e., *class*(*S_i_*) = (*CH*). A sensor node also becomes a CH if it cannot find any CHs from the first time.

When a sensor node becomes a CH, it selects one of its available channels as its operating channel randomly, transmits beacons on the *C_control_*, waits for any join packet, and responds to the join packet with information about its operating channel. The randomly selected operating channel is a temporary one because we have not proceeded to the spectrum decision module. We include the temporary operating channel as a basic requirement in that both parties (CH and CM) find the operating channel as a vacant channel. At this stage, the CH type is type 0 (zero), i.e., *type*(*CH*)*_i_* = 0. (Explanations about CH type are in the next section.) The outcome of the clustering algorithm is that each sensor node has selected its class as either a CH or a CM.

#### 3.2.3. Spectrum Decision Module of C Mode

In the spectrum decision module, the CHs are responsible for selecting an operating channel and a backup channel, and managing their CMs. The selected operating and backup channels may be licensed channels or unlicensed channels. There are four tasks in the spectrum decision module: spectrum characterization, spectrum selection, CM coordination, and spectrum access.

All sensor nodes (CHs and CMs) perform spectrum characterization. Although CMs are not involved in spectrum selection, they need to characterize the spectrum in order to update the spectrum history, which they will use if they become CHs on the next frame. The input of spectrum characterization is the outcome of the spectrum sensing module. The sensor nodes characterize the licensed channels and the SNR calculation for licensed channels (*SNR_x_*) is as follows:

(3) $$SNR_{x}={\left(P_{PUsignal}\right)}_{x}/{\left(P_{PUnoise}\right)}_{x}$$

where (*P_PUsignal_*)*_x_* is the power received from the closest PU on licensed channel *A_x_* inside the sensor node’s transmission range, (*P_PUnoise_*)*_x_* is the power received from all PUs except for the closest one inside its interference range.

We determine that the best *SNR_x_* is equal to 0/0, which means that there is no PU at all. The next best *SNR_x_* is equal to 0/*V* (*V* is an arbitrary value), which means that all PUs are outside the sensor node’s transmission range, even though there are PUs inside its interference range. In our algorithm, we replace the value 0/0 to 1 (one) and 0/*V* to 0 (zero). Using the SNR value, the status of the licensed channel is updated. If *SNR_x_* = 1, then:

(4) $$Status{\left(A_{x}\right)}_{i}=(idle)$$

otherwise:

(5) $$Status{\left(A_{x}\right)}_{i}=(busy)$$

**Figure 3:**
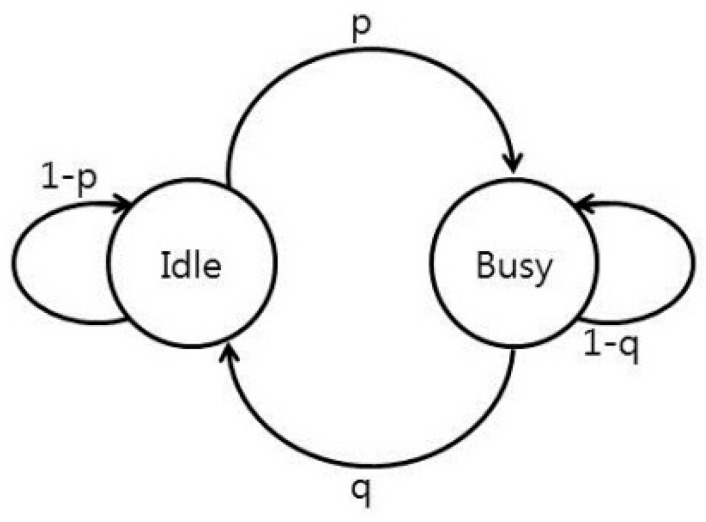
Markov chain for a licensed channel (*p* = probability that an idle channel becomes busy, *q* = probability that a busy channel becomes idle).

We include a Markov chain to predict the channels’ status and to update the channel holding time. Channel holding time is defined as the expected “time” that the channel will remain idle for licensed channels or clean for unlicensed channels (“time” refers to the number of frames.) We created a Markov chain for each licensed channel, as shown in Figure 3.

Markov chains are updated based on the spectrum characterization status. The value (1 − *p*) is the probability of an idle channel to remain idle and it is considered to calculate the channel holding time as follows: If (1 − *p*)*^t^* > *p_thres_*, *t* ≥ 1, and *t_max_* ≥ 1, then:

(6) $${\left(CHT_{x}\right)}_{f}={\left(CHT_{x}\right)}_{fprev}+\sum_{t=1}^{t_{\max}}1$$

where (*CHT_x_*)*_f_* and (*CHT_x_*)*_fprev_* are the channel holding times on channel *A_x_* for the current frame and the previous frames, respectively. The term (1 − *p*)*^t^* is the probabilities that an idle channel will remain idle for time *t*, *p_thres_* is a predetermined probability, and *t_max_* is the highest integer for which the statements (1 − *p*)*^t^* > *p_thres_* is still valid. If (1 − *p*) < *p_thres_*, then:

(7) $${\left(CHT_{x}\right)}_{f}=\frac{1}{2}{\left(CHT_{x}\right)}_{fprev}$$

If *p* = 0, then:

(8) $${\left(CHT_{x}\right)}_{f}={\left(CHT_{x}\right)}_{fprev}+CHT_{\max}$$

where *CHT_max_* is a predetermined constant.

Spectrum selection tasks are performed only by the CHs. CMs go to sleep to save energy during spectrum selection tasks, and they wake up to receive configuration settings at the beginning of CM coordination tasks. In C mode, the outcome is the selection of one operating channel and one backup channel.

CHs are divided into four types depending on the number of idle licensed channels. The CH types are:

(9) $$Type{\left(CH\right)}_{i}=\{\begin{array}{cc} 1 & \text{if}|{\left(A_{idle}\right)}_{i}|\;=0\\ 2 & \text{if}|{\left(A_{idle}\right)}_{i}|\;=1\\ 3 & \text{if}|{\left(A_{idle}\right)}_{i}|\;=2\\ 4 & \text{if }2<\;|{\left(A_{idle}\right)}_{i}|\;\leq L \end{array}$$

where type(*CH*)*_i_* is the CH type of *S_i_*, and (*A_idle_*)*_i_* is the set of idle licensed channels observed by *S_i_*, i.e.,:

(10) $$\begin{array}{ll} {\left(A_{idle}\right)}_{i} & ={\left\{A_{x1},A_{x2},...,A_{xl}\right\}}_{i}\\ & \;={\left\{A_{xv}|A_{xv}\in A,status(A_{xv})=(idle),\;1\leq v\leq L\right\}}_{i} \end{array}$$

where *v* is an arbitrary value, 1 ≤ *v* ≤ *L*, and *A_x1_* ≠ *A_x2_* ≠ … ≠ *A_xl_*.

For *S_i_* with *type*(*CH*)*_i_* = 1, because there is no idle licensed channel, both the operating channel and backup channel for the current frame, (*C_i_*)*_f_* and (*C’_i_*)*_f_*, are selected from a set of unlicensed channels. Hence:

(11) $$\left[{\left(C_{i}\right)}_{f},{\left(C\prime_{i}\right)}_{f}]=[{\left(C_{i}\right)}_{f}\in {\left(B_{clean}\right)}_{i},\;{\left(C\prime_{i}\right)}_{f}\in \{{\left(B_{clean}\right)}_{i}-{\left(C_{i}\right)}_{f}\}\right]$$

for 1 ≤ *i* ≤ *N* and class(*S_i_*) = (*CH*), where the set (*B_clean_*)*_i_* is the clean unlicensed channels of *S_i_*, i.e.,:

(12) $$\begin{array}{ll} {\left(B_{clean}\right)}_{i} & ={\left\{B_{y1},B_{y2},...,B_{yk}\right\}}_{i}\\ & \;={\left\{B_{yv}|B_{yv}\in B,status(B_{yv})=(clean),\;1\leq v\leq K\right\}}_{i} \end{array}$$

where *v* is an arbitrary value, 1 ≤ *v* ≤ *K*, and *B_y1_* ≠ *B_y2_* ≠…≠ *B_yk_*.

For *S_i_* with *type*(*CH*)*_i_* = 2, the operating channel is set to be the only idle licensed channel, and the backup channel is selected from (*B_clean_*)*_i_*. For *S_i_* with *type*(*CH*)*_i_* = 3, one of its idle licensed channels is set as the operating channel, and the remaining one idle licensed channel is set as the backup channel. Hence:

(13) $$\left[{\left(C_{i}\right)}_{f},{\left(C\prime_{i}\right)}_{f}]=[A_{x1},{\left(C\prime_{i}\right)}_{f}\in {\left(B_{clean}\right)}_{i}\right]$$

for *type*(*CH*)*_i_* = 2, and:

(14) $$\left[{\left(C_{i}\right)}_{f},{\left(C\prime_{i}\right)}_{f}]=[A_{x1},A_{x2}]\;or\;[A_{x2},A_{x1}\right]$$

for *type*(*CH*)*_i_* = 3.

Additionally, for *S_i_* with *type*(*CH*)*_i_* = 2 or *type*(*CH*)*_i_* = 3, the *S_i_* broadcasts the licensed channel selection on the *C_control_*. For *S_i_* with *type*(*CH*)*_i_* = 4, the *S_i_* performs spectrum etiquette by allowing *S_i_* with *type*(*CH*)*_i_* = 2 or *type*(*CH*)*_i_* = 3 to claim their operating channel first. Thus, *S_i_* with *type*(*CH*)*_i_* = 4 listens for broadcasts on the *C_control_* before it selects its operating channel. After listening to some declarations of operating channels, *S_i_* with *type*(*CH*)*_i_* = 4 eliminates the idle channels that have been claimed, and selects its operating and backup channels. Hence:

(15) $$\left[{\left(C_{i}\right)}_{f},{\left(C\prime_{i}\right)}_{f}]=[{\left(C_{i}\right)}_{f}\in \{{\left(A_{idle}\right)}_{i}-{\left(A_{excl}\right)}_{i}\},\;{\left(C\prime_{i}\right)}_{f}\in \{{\left(A_{idle}\right)}_{i}-{\left(A_{excl}\right)}_{i}-{\left(C_{i}\right)}_{f}\}\right]$$

where the set (*A_exclude_*)*_i_* contains the idle channels of *S_i_* that have been claimed by other CHs, i.e.,

(16) $$\begin{array}{ll} {\left(A_{excl}\right)}_{i} & ={\left\{{\left(C_{h1}\right)}_{f},{\left(C_{h2}\right)}_{f},...,{\left(C_{h(R-1)}\right)}_{f}\right\}}_{i}\\ & \;=\{\sum_{r=1}^{R-1}{\left(C_{hr}\right)}_{f}\} \end{array}$$

where *R* is the number of total CHs in the network, and (*C_hr_*)_f_ is the operating channel of CH *r* for current frame *f*.

We propose two spectrum selection algorithms: random selection (EGSD-R) and game-theory-based selection (EGSD-G). One of the spectrum selection algorithms is performed to select an operating channel and backup channel, as outlined in Equations (22), (24) and (26). In other words, one of the spectrum selection algorithms is performed by CH type 1, 2, and 4.

In EGSD-R, the operating and backup channels are selected randomly. For example, *S_i_* with *type*(*CH*)*_i_* = 1 selects its operating channel and backup channel randomly from the set of clean unlicensed channels, but it must not be the same as the operating channel. Notice that, although the channels are selected randomly, they are selected inside the set of idle licensed channels or clean unlicensed channels. EGSD-R refines Equations (11), (13) and (15) into:

(17) $$[{\left(C_{i}\right)}_{f},{\left(C\prime_{i}\right)}_{f}]=\{\begin{array}{cc} \left[B_{yu1},B_{yu2}\right] & \text{if }type{\left(CH\right)}_{i}=1\\ \left[A_{x1},B_{yu1}\right] & \text{if }type{\left(CH\right)}_{i}=2\\ \left[A_{xv1},A_{xv2}\right] & \text{if }type{\left(CH\right)}_{i}=3\\ \left[A_{xw1},A_{xw2}\right] & \text{if }type{\left(CH\right)}_{i}=4 \end{array}$$

In Equation (33), the following conditions should be satisfied:

(18) $$(B_{yu1},B_{yu2})\in {\left(B_{clean}\right)}_{i}$$

(19) $$(A_{x1},A_{xv1},A_{xv2})\in {\left(A_{idle}\right)}_{i}$$

and:

(20) $$\left(A_{xw1},A_{xw2})\in \{{\left(A_{idle}\right)}_{i}-{\left(A_{excl}\right)}_{i}\right\}$$

where the values of (*u1*, *u2*, *v1*, *v2*, *w1*, *w2*) are random values with boundaries of 1 ≤ *u1*, *u2* ≤ *yk*, 1 ≤ *v1*, *v2* ≤ *xl*, and 1 ≤ *w1*, *w2* ≤ *h*(*R −* 1). For values *yk*, *xl*, and *h*(*R −* 1), see Equations (21), (23) and (27).

In EGSD-G, we proposed a game theory solution for the spectrum selection problem, which we called mixed strategy with lowest payoff elimination (LPE). The payoff is the channel holding time obtained from the Markov chain in the spectrum characterization stage. First, the payoff is sorted in a descending manner, with the top as the highest payoff:

(21) $$\begin{array}{lll} {payoff}_{i} & = & {\left\{{payoff}_{x},payoff\prime_{y}\right\}}_{i}\\ & = & \{sort({\left({CHT}_{x}\right)}_{f}),sort({\left(CHT\prime_{y}\right)}_{f})|({CHT}_{x1}\geq {CHT}_{x2}\geq ...\geq {CHT}_{xl}),\\ & & (CHT\prime_{y1}\geq CHT\prime_{y2}\geq ...\geq CHT\prime_{yk}){\}}_{i} \end{array}$$

where *payoff_i_* is the payoff of *S_i_*, *payoff_x_* and *payoff’_y_* are the payoff of licensed channels and unlicensed channels, respectively, observed by *S_i_*. (The variable CHT written without frame information is CHT at the current frame, i.e., *CHT_x1_* = (*CHT_x1_*)*_f_*, *CHT_x1_* ≠ (*CHT_x1_*)*_fprev_*.) Then, we perform lowest-payoff elimination by deleting the channel with payoff lower than half of the maximum payoff:

(22) cutpayoffi={cutpayoffx,cutpayoff′y}i    ={(CHTx1,CHTx2,...,CHTxlpe|CHTxlpe≥12CHTx1,xlpe≤xl),      (CHT′y1,CHT′y2,...,CHT′ylpe| CHT′ylpe≥12CHT′y1,ylpe≤yk)}i

where *cutpayoff_i_* is the payoff without the eliminated payoffs, and the payoffs for licensed channels and unlicensed channels are stored in *cutpayoff_x_* and *cutpayoff’_y_*, respectively, observed by *S_i_*. The remaining channels on *cutpayoff_i_* are called the candidate channels. Between these candidate channels, an operating channel and a backup channel are selected. As the payoff of a channel is higher, the probability of it getting selected as an operating/backup channel is higher.

Channel selection depends on a probability distribution called selection game probability (SGP). The SGP of *S_i_* is (*SGP*)*_i_* = {(*SGP*)*_x_*, (*SGP’*)*_y_*}*_i_*, where (*SGP*)*_x_* and (*SGP’*)*_y_* are the SGPs for licensed channels and unlicensed channels, respectively. Thus:

(23) $${\left(SGP\right)}_{x}=\{SGP(A_{x1}),SGP(A_{x2}),...,SGP(A_{xlpe})|SGP(A_{x1})\geq SGP(A_{x2})\geq ...\geq SGP(A_{xlpe})\}$$

(24) $${\left(SGP\prime \right)}_{y}=\{SGP\prime (B_{y1}),...,SGP\prime (B_{ylpe})|SGP\prime (B_{y1})\geq SGP(B_{y2})\geq ...\geq SGP\prime (B_{ylpe})\}$$

where *SGP*(*A_xv_*) and *SGP’*(*B_yv_*) are the probability of channel *A_xv_* and *B_yv_* being selected as the operating or backup channel, respectively (*v* is arbitrary value). Moreover, *SGP*(*A_x1_*) + *SGP*(*A_x2_*) + … + *SGP*(*A_xlpe_*) = 1, as well as *SGP’*(*B_y1_*) + *SGP’*(*B_y2_*) + … + *SGP’*(*B_ylpe_*). The formulation of the EGSD-G spectrum selection is similar to that of EGSD-R, as shown in Equation (33). However, the conditions are different, i.e.,:

(25) $$[{\left(C_{i}\right)}_{f},{\left(C\prime_{i}\right)}_{f}]=\{\begin{array}{cc} \left[B_{yu1},B_{yu2}\right] & \text{if }type{\left(CH\right)}_{i}=1\\ \left[A_{x1},B_{yu1}\right] & \text{if }type{\left(CH\right)}_{i}=2\\ \left[A_{xv1},A_{xv2}\right] & \text{if }type{\left(CH\right)}_{i}=3\\ \left[A_{xv1},A_{xv2}\right] & \text{if }type{\left(CH\right)}_{i}=4 \end{array}$$

for 1 ≤ *i* ≤ *N*, class(*S_i_*) = (*CH*), operation mode is C mode, and the selection algorithm is EGSD-G, where:

(26) $$(B_{yu1},B_{yu2})\in {\left({cutpayoff\prime }_{y}\right)}_{i}$$

(27) $$(A_{x1},A_{xv1},A_{xv2})\in {\left({cutpayoff}_{x}\right)}_{i}$$

and the selection of (*B_yu1_*, *B_yu2_*, *A_xv1_*, *A_xv2_*) follows their respective probabilities (*SGP*)*_x_* and (*SGP*)*_y_* of *S_i_*, as in Equations (23) and (24). For *S_i_* with *type*(*CH*) = 3, *CHT_xv1_* ≥ *CHT_xv2_*.

After the CHs perform spectrum selection (either EGSD-R or EGSD-G), they continue to carry out CM coordination tasks. The CH begins by informing the sink and their respective CMs about the selected operating channel and backup channel via *C_control_* and via the temporary operating channel, respectively. The sink collects the operating channel and backup channel information from all CHs and creates an inter-cluster data transmission schedule (IE-DTS). The sink considers the remaining time slots of the current frame and divides the time slots among the number of different operating channels selected by the CHs (the value of time slots for data transmission is fixed, and it depends on the operation mode). The sink assigns a disjoint time slot for each different channel; however, the CHs that select the same operating channel are assigned to the same time slot. IE-DTS contains the time slot and channel pairs, which are sent back to the CHs. Meanwhile, the CMs, upon receiving the information about the operating and backup channels, check on the operating channel’s availability status on their side (notice that the availability requirement is looser than the idle requirement). If the CMs find out that the assigned operating channel is stated as available, then they send an acknowledgement back to their respective CH on the assigned operating channel. Otherwise the CM goes into sleep state until the end of the frame and sends a normal C-mode request to the sink.

**Figure 4:**
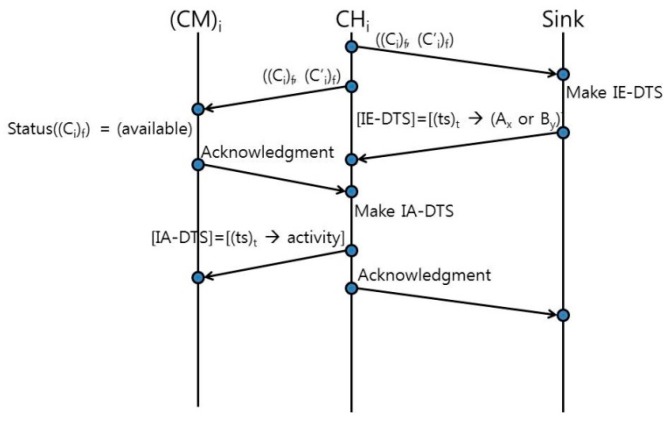
CM coordination tasks ((*ts*)*_t_* means time slot *t*).

The CH receives the IE-DTS from the sink on the *C_control_* and receives some acknowledgements from its CMs on the operating channel. Upon receiving the IE-DTS from the sink, each CH extracts its own operating channel and its determined schedule. Then each CH creates its own intra-cluster data transmission schedule (IA-DTS) and determines the appropriate time slot for data collection activities from its CMs. The data collection activities include environment sensing, data transmission to the CH, and going into sleep state. The IA-DTS contains the time slot and data collection activity pairs, and it is sent to the CMs. The CMs receive IA-DTS from their CHs and set their timers to the scheduled data collection activities. Lastly, the CHs send an acknowledgement to the sink. The tasks in CM coordination are shown in Figure 4.

Both CHs and CMs perform spectrum access by reconfiguring their transmission power. The CMs reconfigure their transmission power so that minimum power is required in order to send data to their CHs. The CHs reconfigure their transmission power so that minimum power is required in order to send data to the sink.

#### 3.2.4. Data Transmission Module of C Mode

The data transmission in this module is the environment-sensing data collection (not spectrum sensing results). The data transmissions from the CMs to the CHs follow the IA-DTS, whereas the data transmissions from the CHs to the sink follow the IE-DTS. However, after we conducted a number of simulations, we noticed that sometimes the operating channels selected by the CHs were mostly the same, particularly when the idle licensed channels were limited. In that case, the sink received only a few different sets of operating channels from a larger set of CHs. In other words, multiple CHs were selecting the same operating channels. If we allow this kind of configuration, the sink would end up creating IE-DTS where the intervals of data transmission of each CH were brief, not allowing the CMs to go to sleep state. Therefore, we included a threshold for a minimum active channel. If the set of operating channels reported by the CHs is less than the minimum active channel, then the sink inserts some gaps between data collections.

### 3.3. Data Transmission Mode (D Mode)

#### 3.3.1. Spectrum Sensing Module of D Mode

The spectrum sensing module of D mode exploits partial spectrum sensing (PSS), in which the sensor nodes perform spectrum sensing only on their operating channels and backup channels selected from a previous frame. In the D mode, the clustered topology is preserved and the sensor nodes are either cluster heads (CHs) or cluster members (CMs).

Firstly, CMs check the connectivity to their CHs by sending a control packet. Meanwhile, the CHs examine whether the backup channel is empty or not. When the CHs receive the control packets from their respective CMs, CHs reply with an acknowledgement and the status of the backup channel. After control packets exchanges are finished, CHs and CMs perform PSS. If there is no PU transmission detected by sensor node *S_i_* on its operating channel selected from the previous frame ((*C_i_*)*_fprev_*), then:

(28) $$Status{\left(C_{i}\right)}_{fprev}=(available)$$

otherwise:

(29) $$Status{\left(C_{i}\right)}_{fprev}=(obsolete)$$

The backup channel is sensed when it is not empty, i.e., backup channel selected from the previous channel (*C’_i_*)*_fprev_* ≠ Ø. Similarly, if there is no PU transmission detected by sensor node *S_i_* on (*C’_i_*)*_fprev_*, then:

(30) $$Status{\left(C\prime_{i}\right)}_{fprev}=(available)$$

otherwise:

(31) $$Status{\left(C\prime_{i}\right)}_{fprev}=(obsolete)$$

The CMs send the spectrum-sensing results to their CH. As the CRSN continues to operate, the sensor nodes eventually deplete its energy and become inactive. If a CH becomes inactive, its CMs would not receive acknowledgement packets; thus, they would go to sleep and wake up at the last time slot of the current frame to send urgent requests for C mode because an inactive CH causes the entire cluster to be inactive. If a CH does not receive any control packet (all of its CMs are inactive), it proceeds to the next activities and modules, and also sends a normal request for C mode at the last time slot. Aside from the energy depletion of the sensor nodes, the operation channel’s quality degradation also causes the CHs or CMs to be unable to receive packets from each other. However, our proposed algorithm does not differentiate those causes of failed transmissions.

**Figure 5:**
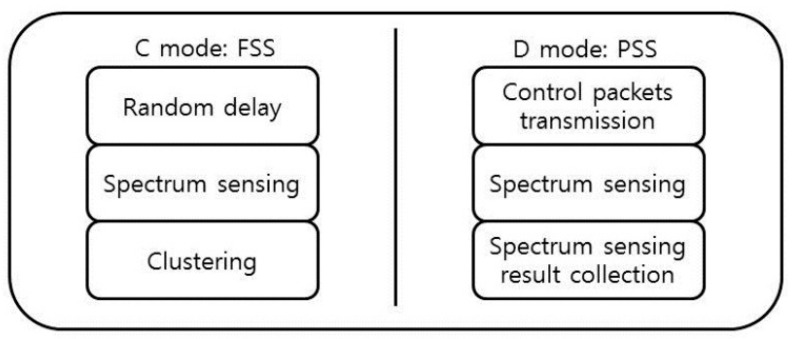
Spectrum sensing module.

The outcomes of PSS are a list of operating channels and backup channels, their status (available or obsolete), and the variables to calculate the SNR. The activities of spectrum sensing modules, both FSS and PSS, are shown in Figure 5.

#### 3.3.2. Spectrum Decision Module of D Mode

The tasks in spectrum decision module of D mode are similar with C mode, except that in D mode some of the tasks are shorter because the sensor nodes update the information regarding two channels of operating and backup channels.

All sensor nodes (CHs and CMs) perform spectrum characterization, and the underlying tasks are the same as in C mode. However, in D mode, sensor nodes only characterize the operating channel and backup channel. The SNR calculation for unlicensed channels (*SNR’_y_*) is as follows:

(32) $$SNR\prime_{y}=P_{highestnoise}-{\left(P_{noise}\right)}_{y}$$

where *P_highestnoise_* is the maximum noise level, and (*P_noise_*)*_y_* is the actual noise level on channel *B_y_*. (*SNR’_y_* is not actually a ratio.)

The channel status update is as follows: if *SNR’_y_* is higher than a predetermined threshold, *SNR’_thres_*, then:

(33) $$Status{\left(B_{y}\right)}_{i}=(clean)$$

otherwise:

(34) $$Status{\left(B_{y}\right)}_{i}=(noisy)$$

Spectrum sensing on unlicensed channels are not performed unless the channels ever become an operating or backup channel (operating/backup channel sensing is performed in partial spectrum sensing). Thus, for other licensed channels:

(35) $$Status{\left(B_{y}\right)}_{i}=(unknown)$$

**Figure 6:**
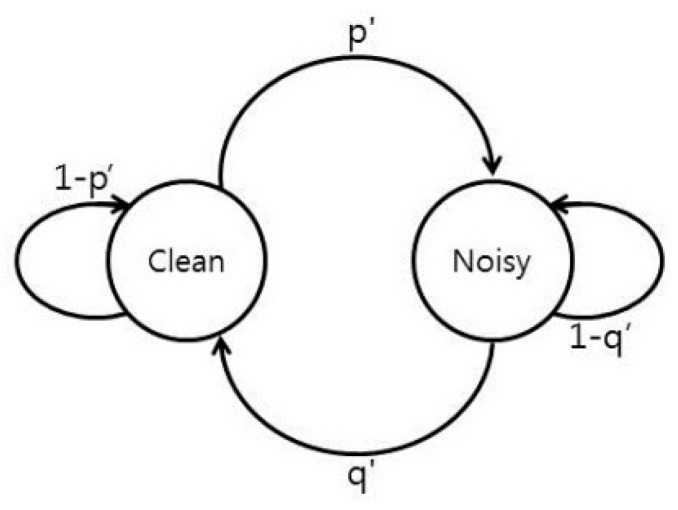
Markov chain for an unlicensed channels (*p’* = probability that a clean channel becomes noisy, and *q’* = probability that a noisy channel becomes clean).

In case of unlicensed channels, channel holding time is defined as the expected “time” that the channel will remain clean. Similarly, a Markov chain is created for each unlicensed channel, as shown in Figure 6.

Only the Markov chains for operating and backup channels are updated. The value (1 − *p’*) is the probability of a clean channel to remain clean and it is considered to calculate the channel holding time as follows: If (1 − *p’*)*^t^* > *p’_thres_*, *t* ≥ 1, and *t_max_* ≥ 1, then:

(36) $${\left(CHT\prime_{y}\right)}_{f}={\left(CHT\prime_{y}\right)}_{fprev}+\sum_{t=1}^{t_{\max}}1$$

where (*CHT’_y_*)*_f_* and (*CHT’_y_*)*_fprev_* are the channel holding times on channel *B_x_* for the current frame and the previous frames, respectively. The term (1 − *p’*)*^t^* is the probabilities that an idle channel will remain idle or a clean channel will remain clean, for time *t*, *p’_thres_* is a predetermined probabilities, and *t_max_* is the highest integer for which the statements (1 − *p’*)*^t^* > *p’_thres_* is still valid. If (1 − *p’*) < *p’_thres_*, then:

(37) $${\left(CHT\prime_{y}\right)}_{f}=\frac{1}{2}{\left(CHT\prime_{y}\right)}_{fprev}$$

If *p’* = 0, then:

(38) $${\left(CHT\prime_{y}\right)}_{f}={\left(CHT\prime_{y}\right)}_{fprev}+CHT\prime_{\max}$$

where *CHT’_max_* is a predetermined constant.

Similarly, in D mode, spectrum selection tasks are performed only by the CHs. However, the outcome of spectrum selection in D mode is a decision whether the current operating channel can be used or needs to be changed.

Spectrum selection in D mode also divides the CHs into four types depending on the conditions of the operating channel and the backup channel. The CH types are:

(39) $$Type{\left(CH\right)}_{i}=\{\begin{array}{cc} 1 & \begin{array}{l} \text{if }status{\left(C_{i}\right)}_{fprev}=(available)\;\\ \text{and }status{\left(C\prime_{i}\right)}_{fprev}=(available) \end{array}\\ 2 & \begin{array}{l} \text{if }status{\left(C_{i}\right)}_{fprev}=(available)\;\\ \text{and }status{\left(C\prime_{i}\right)}_{fprev}=(obsolete) \end{array}\\ 3 & \begin{array}{l} \text{if }status{\left(C_{i}\right)}_{fprev}=(obsolete)\;\\ \text{and }status{\left(C\prime_{i}\right)}_{fprev}=(available) \end{array}\\ 4 & \begin{array}{l} \text{if }status{\left(C_{i}\right)}_{fprev}=(obsolete)\;\\ \text{and }status{\left(C\prime_{i}\right)}_{fprev}=(obsolete) \end{array} \end{array}$$

where the values of *status*(*C_i_*)*_fprev_* and (*C’_i_*)*_fprev_* are obtained from Equations (28)–(31).

For *S_i_* with *type*(*CH*)*_i_* = 1, because both channels remain available, new channel selection is not needed. That is:

(40) $$\left[{\left(C_{i}\right)}_{f},{\left(C\prime_{i}\right)}_{f}]=[{\left(C_{i}\right)}_{fprev},{\left(C\prime_{i}\right)}_{fprev}\right]$$

For *S_i_* with *type*(*CH*)*_i_* = 2, the backup channel becomes empty. Similarly, for *S_i_* with *type*(*CH*)*_i_* = 3, the backup channel becomes the operating channel and the backup channel becomes empty. The *S_i_* with *type*(*CH*)*_i_* = 2 or *type*(*CH*)*_i_* = 3 will send a normal request of C mode to the sink. Thus:

(41) $$\left[{\left(C_{i}\right)}_{f},{\left(C\prime_{i}\right)}_{f}]=[{\left(C_{i}\right)}_{fprev},(\varphi )\right]$$

for *type*(*CH*)*_i_* = 2 (*φ* indicates an empty set), and:

(42) $$\left[{\left(C_{i}\right)}_{f},{\left(C\prime_{i}\right)}_{f}]=[{\left(C\prime_{i}\right)}_{fprev},(\varphi )\right]$$

for *type*(*CH*)*_i_* = 3.

For *S_i_* with *type*(*CH*)*_i_* = 4, because both channels have become obsolete, S_i_ has nothing else to do but to request an urgent C mode to the sink:

(43) $$\left[{\left(C_{i}\right)}_{f},{\left(C\prime_{i}\right)}_{f}]=[(\varphi ),(\varphi )\right]$$

The proposed spectrum selection algorithms, EGSD-R and EGSD-G, are not performed in D mode. Next, the CM coordination tasks and spectrum access tasks are identical with those of C mode.

#### 3.3.3. Data Transmission Module of D Mode

The tasks for the data transmission modules for D mode are identical with those of C mode.

## 4. Performance Evaluation

**Figure 7:**
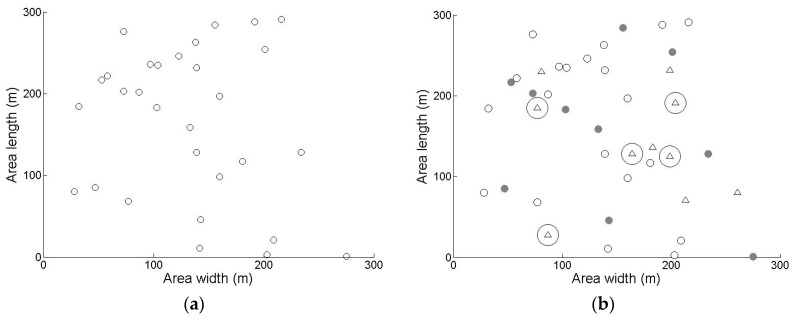
Network topology of (**a**) sensor nodes only and (**b**) sensor nodes after clustering and PUs.

We evaluated the performance of EGSD-R and EGSD-G and compared the results with MVSD (see Section 2). The tool we used is MATLAB with the simulation settings presented in Table 1. The network topology is shown in Figure 7. We selected two evaluation parameters: network lifetime, defined as the time until half of the sensor nodes are alive; and overhead, defined as number of time-slot spends for coordination divided by the total number of time slots.

**Figure 8:**
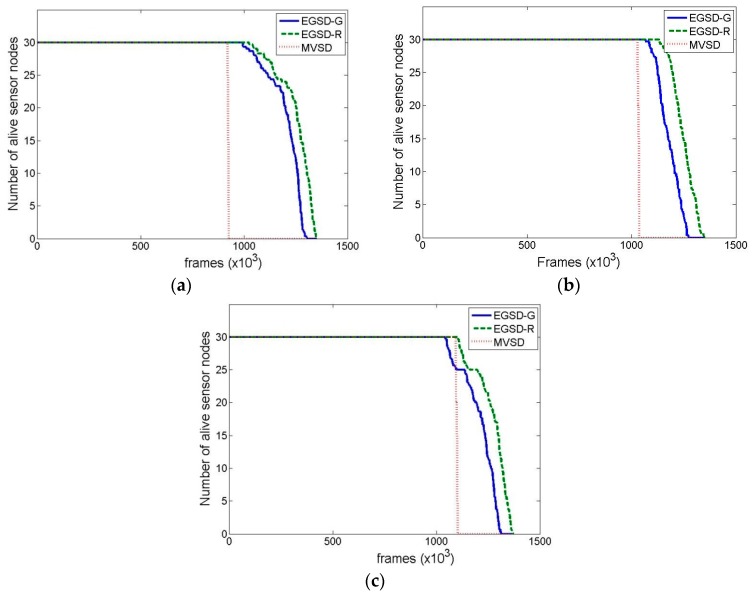
Network lifetime when the maximum number of PUs is (**a**) 30; (**b**) 60; and (**c**) 90, where PUs do not have a favorite channel.

Figure 8 shows the network lifetime results with a varied number of maximum PUs. The lifetimes of EGSD-G and EGSD-R are relatively consistent, whereas the lifetimes of MVSD increase with an increasing maximum number of PUs. These results show the merit of a centralized algorithm where the central entity has global knowledge of the network and is therefore able to optimize network performance even when the number of PUs increases. However, both EGSD-G and EGSD-R outperform MVSD. The reason is that MVSD requires multiple control-packet exchanges to the sink for each frame, while EGSD requires fewer transmissions of control packets, especially in the D mode. On average, EGSD-R and EGSD-G have 25.5% and 19.8% longer lifetimes, respectively, compared with MVSD. The best performance is obtained when the number of PUs is 30.

Despite the additional complexities of EGSD-G com-pared with EGSD-R, the performance of the latter turned out to be better. EGSD-R outperforms EGSD-G by 4.6%, on average. We found out that this occurred because the PUs were also selecting their channels randomly. Random channel selection of PUs renders prediction by a Markov chain less optimal. Therefore, we include another scenario where the PUs have a certain favorite channel and would likely select it as their operating channel. Notice that this selection is not fixed but probabilistic, i.e., the PUs do not always select the favorite channel on each occasion. We consider that this assumption is acceptable especially if the PUs are TV viewers, who may have some preferred TV shows during certain times.

**Figure 9:**
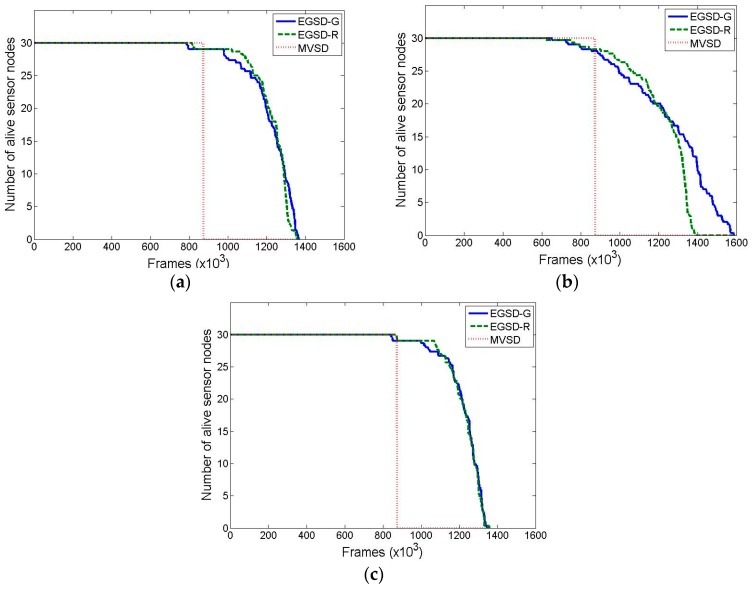
Network lifetime when the maximum number of PUs is (**a**) 30; (**b**) 60, and (**c**) 90, where PUs have a favorite channel.

The results of the network lifetime where the PUs have favorite channels are shown in Figure 9. We expected the performance of EGSD-G to be better than the others; however, the simulation results did not fully support our expectation. When the numbers of maximum PU are 30 and 90, the EGSD-G reached almost the same lifetime as EGSD-R, with a minor difference of two and seven frames, respectively. However, when the number of PUs is 60, EDSG-G had 3.2% longer lifetime compared with EGSD-R. Another observation is that the last sensor node depletes its energy after a longer time (14.2% longer) in EGSD-G than in EGSD-R. Therefore, we conclude that the number of PUs affects the performance of EGSD-G relative to EGSD-R. When the number of PUs is low, the prediction by the Markov chain did not perform optimally because there was not enough data. Nevertheless, when the number of PUs was high, the values on the Markov chain tended to be similar. Thus the prediction also did not perform optimally. However, in this scenario, EGSD-G and EGSD-R also outperform MVSD by 46.6% and 44.9%, respectively.

**Figure 10:**
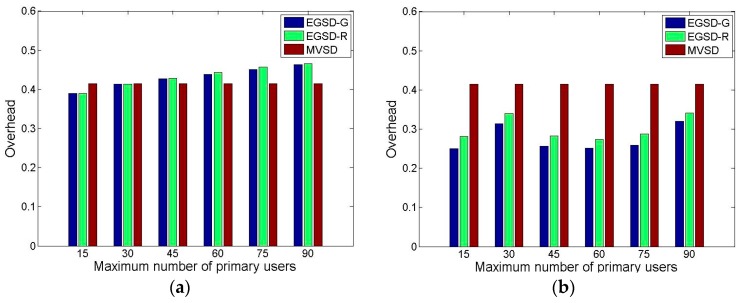
Network overhead when (**a**) PUs do not have a favorite channel and (**b**) PUs have a favorite channel.

We also included a scenario where PUs had a favorite channel in the overhead evaluation. Figure 10 shows the overhead when the numbers of maximum PUs are varied. When PUs have no favorite channel, the overheads of EGSD-G and EGSD-R are similar and increase as the number of PUs increases. Overall, the proposed EGSD has higher overhead than MSDV by 4.9%, on average. We predicted this result because MVSD is a centralized method in which the overhead is fixed, whereas EGSD is a distributed method in which the overhead depends on the network condition. The overhead in EGSD depends on the number of PUs because when the number of PUs increases, the probability of the operating channel becoming obsolete also increases, resulting in more frequent C modes. (In C mode, the time spent on coordination is higher than in D mode.) However, when the PUs have a favorite channel, the EGSD-G and EGSD-R outperform MVSD by 31.7% and 26.8%, respectively. The reason for this improvement is that when the PUs have a favorite channel, the probability of an arbitrary operating channel of a sensor node being claimed by the PUs is lower, except that the operating channel used is the favorite channel. Hence, the portion of D mode is higher than that of C mode. Moreover, in this result, we observe a slight improvement of EGSD-G over EGSD-R. Last but not least, we determine that there is a case where the distributed method may lead to lower overhead than the centralized method.

## 5. Conclusions

In this paper, we have proposed a spectrum decision scheme called EGSD for CRSNs, which supports the two operation modes of coordination mode and data collection mode. The core of EGSD is the two spectrum selection algorithms of random selection called EGSD-R and game-theory-based selection called EGSD-G. The simulation results show that EGSD has longer lifetime and lower overhead compared to the conventional MVSD scheme. In the best scenario, EGSD outperforms MVSD by 46.6% longer lifetime and 31.7% lower overhead. The improvements are mainly because EGSD is a distributed method that requires fewer control packet exchanges to the sink. The other sources of the performance improvement are the simple yet energy-aware clustering method, the predictions by a Markov chain (for EGSD-G), and a data collection mode that consumes less energy than the coordination mode. We have also observed that EGSD-G performs slightly better than EGSD-R. In future works, we intend to: (1) combine the game theory with other machine-learning techniques to exploit the spectrum usage pattern of PUs; (2) incorporate real measurements of PU spectrum usage; and (3) consider application-specific sensor placements. In addition, the situation that PUs have a favorite channel will be taken into consideration.

## Figures and Tables

**Table 1:** Simulation Parameters.

Parameter	Value
Network area	300 m × 300 m
Number of sensor nodes	30 nodes
Installation method	Random
Number of sink	1
Location of sink	150 m, 150 m
PU protection range	50 m
PU active probability = PU passive probability	0.5
PU location mobility = PU channel mobility	0.5
Number of licensed channel	29 channels
Number of unlicensed channel	29 channels
Maximum noise level	10
Maximum peak interference	14
*SNR’_thres_*	5
Common control channel frequency	474 MHz
Licensed channel frequencies	482–546 MHz (bandwidth 8 MHz) and 536–787 MHz (bandwidth 13 MHz)
Unlicensed channel frequencies	ISM 2.4 GHz and 5 GHz
Power supply of sensor nodes	9360 J × 2 [48,49]
Sensing range	50 m
Transmission range (initial)	100 m
Interference range	150 m
Maximum limit of random delay	25 time slots
Residual energy levels	5 levels
*p_thres_* = *p’_thres_*	0.5
*CHT_max_* = *CHT’_max_*	2
Minimum active channel	5 channels
C mode request threshold	3 normal requests or 1 urgent request
